# Artificial Intelligence Application in Cornea and External Diseases

**DOI:** 10.3390/diagnostics15243199

**Published:** 2025-12-15

**Authors:** Te-Chen Lu, Chun-Hao Huang, I-Chan Lin

**Affiliations:** 1 School of Medicine, College of Medicine, Taipei Medical University, Taipei 110301, Taiwan; b101113069@tmu.edu.tw; 2 Department of Ophthalmology, Wan Fang Hospital, Taipei Medical University, Taipei 116081, Taiwan; 3 Department of Ophthalmology, School of Medicine, College of Medicine, Taipei Medical University, Taipei 110301, Taiwan

**Keywords:** artificial intelligence, corneal disease, keratoconus, dry eye disease, infectious keratitis, pterygium, Fuchs endothelial corneal dystrophy, corneal transplantation

## Abstract

Corneal diseases are a leading cause of blindness worldwide, although their early detection remains challenging due to subtle clinical presentations. Recent advances in artificial intelligence (AI) have shown promising diagnostic performance for anterior segment disorders. This narrative review summarizes current applications of AI in the detection of corneal conditions—including keratoconus (KC), dry eye disease (DED), infectious keratitis (IK), pterygium, Fuchs endothelial corneal dystrophy (FECD), and corneal transplantation. Many AI models report high accuracy on test datasets, comparable to, and in some studies exceeding, that of junior ophthalmologists. In addition to detection, AI systems can automate image labeling and support education and patient home monitoring. These findings highlight the potential of AI to improve early management and standardized classification of corneal diseases, supporting clinical practice and patient self-care.

## 1. Introduction

In recent decades, artificial intelligence (AI) has been increasingly integrated into ophthalmology, enabling advancements in disease detection, progression prediction, and clinical decision support. AI encompasses various computational methods, including machine learning (ML), deep learning (DL), artificial neural networks (ANNs), and deep neural networks (DNNs). These techniques facilitate the processing and analysis of large datasets with high accuracy, establishing AI as an adjunctive tool for diagnostic and prognostic evaluation.

Corneal diseases, including keratoconus (KC), dry eye disease (DED), infectious keratitis (IK), pterygium, and Fuchs endothelial corneal dystrophy, can lead to significant visual impairment and reduced quality of life. Traditionally, diagnosis has relied on clinical signs, slit-lamp examination, functional tests, and ophthalmologists’ interpretation of corneal topography and tomography scans. These diagnostic approaches are limited by variability in corneal map accuracy, dependence on subjective clinical assessment, and the lack of universally accepted diagnostic criteria. Recent advances in AI provide solutions to longstanding diagnostic challenges in corneal disease. When trained on large datasets of corneal images and corresponding clinical data, AI models can identify subtle pathological features that may be overlooked by clinicians. Evidence from studies supports this; for instance, the CorneAI system has been shown to increase ophthalmologists’ diagnostic accuracy from 79.2% to 88.8% [1]. While AI has not yet reliably outdone top clinicians across the board, it works well as a supportive element, boosting both accuracy and efficiency in the process.

**Figure 1:**
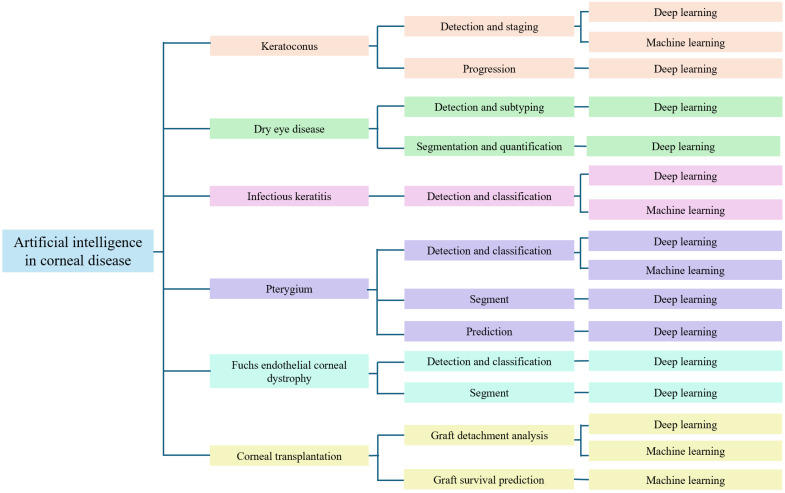
Taxonomy for artificial intelligence in corneal disease.

This narrative review synthesizes recent findings regarding the application of AI in the diagnosis of corneal diseases. The diagnostic performance of various AI models is compared with that of ophthalmologists, emphasizing their potential to improve clinical decision-making in corneal and external eye disease management, as shown in Figure 1.

## 2. Method

A literature review was conducted in PubMed, Google Scholar, Embase, and Scopus from January 1994 to September 2025. The search strategy integrated Medical Subject Headings (MeSH) terms with free-text terms related to artificial intelligence and corneal diseases. These included terms such as “machine learning,” “deep learning,” and “convolutional neural networks,” as well as representative disease names (e.g., “keratoconus,” “infectious keratitis,” “dry eye disease,” and “pterygium”) and relevant imaging modalities covered in this review. Only English-language articles were included. We included original research and review articles. Two reviewers independently checked titles and abstracts for relevance and then reviewed the full texts. The same reviewers assessed the full texts to decide eligibility. Any disagreements were resolved through discussion, reaching agreement on the final list of included studies. Reference lists of included studies were also manually screened. Case reports, conference abstracts, and studies not involving AI applications or lacking performance metrics were excluded.

## 3. Background of Artificial Intelligence Tools

In general diagnosis, ophthalmologists often utilize several AI tools, including ML, DL, ANNs, DNNs, and convolutional neural networks (CNNs). These tools are trained on large datasets of images, including corneal topography maps, anterior segment optical coherence tomography (AS-OCT), slit-lamp images, and more [2]. The steps in developing an AI model are divided into three sections. First, the datasets are separated into three parts, namely training sets, validation sets, and testing sets [3]. Then, we can use training sets to train the AI models and validation sets to check them. Lastly, we can obtain the results from the testing set, which applies to the AI models. AI models can analyze pictures and provide diagnosis results, risk predictions, and support alerts in decision-making.

There are several advantages of AI tools, including high accuracy and precision, reduced measurement error through observation, and increased speed of task completion. However, there are also some disadvantages. For instance, the accuracy of some special cases will become lower because AI models cannot distinguish rare images. Additionally, patient data collected by AI models can easily compromise patient privacy. Therefore, careful data management and consideration of ethical implications are crucial.

## 4. Keratoconus

Characterized by progressive corneal thinning and ectasia, KC induces significant myopia and irregular astigmatism, leading to substantial visual morbidity [4,5]. While current diagnostic protocols integrate Placido topography, AS-OCT, and corneal tomography [6], the identification of early-stage disease, such as subclinical or forme fruste keratoconus (FFKC), remains elusive. Conventional parameters often lack the sensitivity to detect the earliest morphological signs of disease onset.

Early AI approaches included expert systems and statistical models. An initial expert-system study using eight parameters reported over 80% accuracy, sensitivity, and specificity [7]. Expert classifiers initially proved more effective than traditional keratometry or the Rabinowitz–McDonnell test, achieving up to 99% specificity [8]. This led to the adoption of standard ML algorithms (e.g., support vector machine (SVM), linear discriminant analysis (LDA), and random forests (RFs)) for distinguishing subclinical KC from normal eyes [9]. However, these models required manual feature engineering, a labor-intensive process that limited their ability to fully exploit raw corneal data before the advent of deep learning. The performance of these models depended heavily on the operator’s selection of parameters, potentially oversimplifying the complex, non-linear topographical patterns of early-stage disease.

SVM and decision trees (DTs) were employed for the detection of KC, FFKC, and normal eyes. Although the sensitivity and specificity of both AI models were acceptable, comparative analyses revealed that the results for SVM were slightly lower than those for DT, particularly in sensitivity for FFKC and normal eyes [10,11]. LDA and RF were also developed to predict different stages of KC. The accuracy of both models exceeded 93%, although the sensitivity of RF was slightly higher than that of LDA [12]. Overall, these studies suggest that traditional ML approaches laid an important foundation for advanced AI models in KC detection. Collectively, traditional ML approaches demonstrated that non-linear classifiers (like RF) generally outperform LDA in handling complex corneal metrics, yet they still struggle to generalize across different imaging devices without retraining.

After advancements in AI techniques, neural networks (NNs) were introduced and demonstrated significant improvements. The accuracy of NNs applied to seven categories was 80%, while a two-category model achieved 100% accuracy. However, the differences in sensitivity between the two experiments were substantial [13,14]. These studies indicate that early neural networks faced greater challenges when classifying a wider range of categories (multi-class classification) compared to binary screening tasks. Later studies selected different parameters to classify maps into normal, KC, and non-KC conditions, achieving an accuracy of 96.4%, sensitivity of 94.1%, and specificity of 97.6% in the best scenario [15].

With the rapid advancement of AI, deep learning models, particularly CNNs, have been widely employed for detection. AI tools like KeratoDetect and other CNN models can analyze images such as color-coded maps and AS-OCT. Results from various studies show that CNN models perform well in detecting different stages of KC, with performance often comparable to ophthalmologists’ decisions [4,16,17,18]. However, a critical limitation of many deep learning studies is their reliance on single-center, private datasets with limited ethnic diversity. This raises concerns regarding the models’ generalizability, as corneal thickness and curvature norms vary across populations. Furthermore, the “black-box” nature of end-to-end CNNs hinders clinical trust, as these models often lack explainable features to justify the diagnosis.

More recently, multi-source (multimodal) and hybrid AI models have emerged as a key direction for KC detection to address the limitations of unimodal imaging [19]. Multimodal fusion of Placido/tomographic data with AS-OCT-derived epithelial and stromal metrics improves early and suspect KC detection; for example, a large Translational Vision Science and Technology (TVST) study employing a feedforward ANN reported a precision of ≈98–99% for established KC and markedly better performance for suspect KC than topography alone [20]. Integrating biomechanics has further refined diagnostic precision. DL based on biomechanics uses Scheimpflug dynamic corneal deformation video sequences converted into 3-D pseudo-images; a DenseNet-based model achieved an external test Area Under the Curve (AUC) of ≈0.93 with a specificity of ≈98% [21]. The recent literature also highlights the potential of large language models and advanced screening algorithms to synthesize these complex datasets [22]. Beyond diagnosis, prognostic models are being used to gauge the likelihood of disease progression and preoperative ectasia risks. A TVST study employing a fusion network forecasted progression at the initial visit with accuracy ≈0.83, with age and posterior elevation as key contributors [23]. In a Moorfields AJO cohort, age at presentation, *K*_max_, and minimal corneal thickness were the principal determinants of progression risk, whereas candidate genetic variants added little incremental value [24]. Regarding ectasia screening, the refined tomographic–biomechanical index (TBIv2)—a random forest algorithm integrating Scheimpflug tomography with Corvis ST biomechanics—has demonstrated exceptional precision. It reportedly achieved an Area Under the Receiver Operating Characteristic Curve (AUROC) near 0.999 for clinical ectasia and significantly enhanced the detection of highly asymmetric cases in eyes presenting with normal topography (AUROC ≈ 0.94) [25].

However, the transition from algorithmic success to routine Clinical Decision Support Systems (CDSSs) faces substantial hurdles. A primary limitation is the exclusive reliance of most current models on imaging parameters; they frequently overlook non-imaging risk factors—such as eye rubbing, atopy, and systemic history—that are clinically pivotal for differentiating active progressors from stable cases. Hybrid models integrating CNN extraction with SVM or ANN classifiers offer slight advantages but lack broad validation on diverse cohorts [26]. Consequently, establishing robust clinical utility requires a multifaceted approach: enhancing model explainability (explainable artificial intelligence, XAI) to transparency validate decision drivers, integrating imaging data with demographic and symptomatic profiles from Electronic Health Records (EHRs), and ensuring generalizability through extensive testing across varied hardware and ethnicities (Table 1).

## 5. Dry Eye Disease

Classified as aqueous-deficient or evaporative, DED is a pervasive ocular surface condition often driven by Meibomian Gland Dysfunction (MGD). This underlying pathology destabilizes the tear film, altering its lipid structure and hastening evaporation [2]. The disease burden is highest among women and adults over 50 [27], with clinical evaluation centering on metrics like tear meniscus height (TMH), break-up time (TBUT), and corneal fluorescein staining. Diagnosis, however, is often challenging because etiologies and manifestations vary widely and patient-reported symptoms frequently do not correlate with objective signs. To improve detection and risk stratification and to mitigate the subjectivity of manual grading, clinicians increasingly use meibography, AS-OCT, slit-lamp biomicroscopy, and smartphone-based imaging [28].

Meibography serves as a pivotal tool for delineating gland atrophy and quantifying dropout rates. Given the subjectivity of manual grading, deep learning has effectively established itself as the superior methodology for ensuring reproducibility. The performance gap is substantial: CNN architectures have demonstrated segmentation accuracies of 95.6% [29] and have outperformed clinical experts in classifying severe MGD by a wide margin (80.9% vs. 25.9%) [30]. Despite these impressive metrics, clinical scalability is currently hindered by device dependence; models trained largely on proprietary data (e.g., Keratograph) often struggle to generalize across different infrared imaging platforms. Unsupervised learning has also been explored: Li et al. applied SimCLR-based representation learning to classify DED into six subtypes, yielding more consistent grading standards [31].

Slit-lamp and optical coherence tomography (OCT) images contribute complementary information. A CNN-BUT method applied to slit-lamp images achieved 95% specificity and 83% sensitivity for first break-up time estimation [32]. The Smart Eye Camera, a portable slit-lamp, showed a diagnostic accuracy of 0.789 against Asia Dry Eye Society (ADES) criteria, suggesting utility for community and home settings despite slightly lower resolution compared to desktop devices [33]. AS-OCT analysis reached 84.6% accuracy, outperforming the Schirmer test and approximating ocular surface disease index (OSDI) and TBUT performance [34]. Wide epithelial mapping by OCT also demonstrated high sensitivity and specificity for DED diagnosis [35].

AI has further been applied to TMH images and multimodal datasets. Deep learning models analyzing TMH have shown higher diagnostic accuracy than ophthalmologists [36,37]. CNN-based grading of corneal fluorescein staining reduced inter-observer variability and standardized scoring [38]. Smartphone-based TMH analysis shows acceptable accuracy for home monitoring and may facilitate patient self-management [39]. The synergistic application of meibography, slit-lamp imaging, AS-OCT, and smartphone-based platforms serves to standardize assessment protocols, thereby minimizing subjective error and refining diagnostic precision. Evidence from diverse cohorts validates this multimodal approach, where accuracies consistently surpass 80–90%, and sensitivity and specificity estimates reach near-optimal thresholds (95–100%) in controlled environments [40,41].

However, widespread clinical translation is currently stalled by a fundamental limitation: the predominance of proprietary, homogeneous training data. This lack of representativeness casts doubt on the external validity of current models, particularly regarding their ability to generalize across diverse ethnicities and distinct eyelid anatomies. Moreover, contemporary AI frameworks remain heavily predicated on static imaging parameters alone. Realizing robust CDSS capabilities will require a shift toward the multimodal fusion of heterogeneous data streams, alongside transformer-based architectures capable of encoding temporal blink dynamics and tear film stability. A critical frontier involves the assimilation of “non-visual” metadata—spanning patient history, systemic pharmacology, and subjective indices—to accurately demarcate symptomatic DED from subclinical presentations (Table 2).

## 6. Infectious Keratitis

IK, caused by bacteria, fungi, viruses, or parasites, remains a leading cause of corneal blindness when not identified and addressed early [28]. Clinical differentiation is notoriously difficult due to overlapping phenotypic features, necessitating adjunctive diagnostic tools. AI tools, leveraging slit-lamp and in vivo confocal microscopy (IVCM) images, have notably enhanced diagnostic precision in classifying cases [42]. Traditional ML methods effectively stratified keratitis types but were limited by manual feature extraction [43]. Deep learning addressed this by automating the workflow and often outperforms ophthalmologists: CNN-based models achieved 99.3% accuracy for IK detection, 84% for bacteria keratitis (BK) vs. FK discrimination, and 77.5% for filamentous vs. yeast fungi [44,45]. Today, CNNs like DenseNet121 often surpass human experts, reporting accuracies up to 99.3% [46,47]. Unfortunately, because most validation comes from private, homogeneous datasets, the generalizability of these models to other clinics remains uncertain.

IVCM imaging is particularly valuable for FK. DL has effectively outperformed decision tree approaches, with AUCs frequently >0.90. Reported accuracies for fungal vs. other keratitis reached 87–97%, while filamentous vs. yeast differentiation achieved slightly lower but still clinically meaningful performance [48,49,50,51]. Despite these results, the high cost and steep learning curve of IVCM remain barriers to widespread clinical deployment.

Beyond IVCM, AI models can also be applied to accessible sources, such as smartphone images, to diagnose microbial keratitis (MK). The advancement of smartphone images helped overcome the difficulties of remote places through rapid diagnosis and portability. However, the imbalanced data and the continued need for slit-lamp equipment limited the feasibility of using smartphone images as a dataset [52].

Multi-class systems have emerged. The DeepIK framework achieved AUCs > 0.94 for internal validation and 0.88–0.93 in external validation across bacterial, fungal, viral, Acanthamoeba, and non-infectious keratitis [53]. Such external validation underscores the generalizability of modern models. Other CNNs, like AlexNet and Visual Geometry Group Net (VGGNet), when applied to digital single-lens reflex (DSLR) and confocal images, have delivered near-perfect accuracies in select FK datasets—though questions about overfitting linger [54,55,56].

Despite these advancements, clinical adoption faces practical barriers. A major limitation is that most algorithms operate in isolation, analyzing images while blind to the patient’s context—such as contact lens history, ocular trauma, or systemic comorbidities—which is essential for expert diagnosis. Moreover, the robustness of these systems remains unproven when tested against the variability inherent in diverse ethnic populations and heterogeneous imaging platforms. Future research must prioritize (i) integrating multimodal data (images + EHR) to support holistic decision-making and (ii) validating models on publicly available, multicenter datasets to ensure robust clinical utility [57] (Table 3).

## 7. Pterygium

Pterygium is a frequent growth on the ocular surface, composed of fibrovascular tissue that invades the cornea and can hinder vision if it worsens or extends centrally [58]. AI has drawn increasing attention because slit-lamp or smartphone images can aid in screening and prioritizing cases, particularly in resource-limited settings [58,59]. Methods have progressed from manually designed image features and standard classifiers to DL using CNNs. While a number of studies highlight performance comparable to that of clinicians, clinical deployment is limited by phenotypic diversity and the absence of a standardized grading scale [58,59].

Across slit-lamp and handheld/smartphone images, both classical ML and DL approaches report high diagnostic accuracy. For example, an ensemble model identified pterygia requiring surgery with 94.1% accuracy and an AUC of 0.980 [60]. Segmentation networks (PSPNet or U-Net derivatives) generate lesion masks that support corneal encroachment measurement and preoperative planning [61], and a subsequent framework demonstrated longitudinal tracking of lesion change [62]. A two-stage smartphone-oriented pipeline—Faster R-CNN (ResNet101) for detection and an SE-ResNeXt50-based U-Net for segmentation—achieved high detection accuracy with consistent performance across different smartphone brands, indicating feasibility for mobile deployment [63].

External evaluations are encouraging. Systems trained to detect any pterygium and referable (surgically significant) pterygium reported AUCs of approximately 99.1–99.7% and 98.5–99.7%, respectively, with sensitivities and specificities commonly >90%, including on handheld camera datasets—supporting potential use in community screening and referral pathways [64].

Applications extend beyond color photographs. ML analyses have identified differentially expressed genes and immune microenvironmental features that may serve as biomarkers or therapeutic targets [65]. Clinically, DL-based grading on slit-lamp photographs has been explored for recurrence prediction (with moderate sensitivity in some cohorts) [66]. In addition, classical models using clinical variables can classify postsurgical best-corrected visual acuity (BCVA) change with high accuracy (e.g., support vector machine accuracy 94.44%, sensitivity 92.14%, specificity 100%) [67].

The integration of multimodal and large-language models (LLMs) marks a distinct frontier in diagnostic tooling. For instance, a smartphone-based framework using a GPT-4-class model yielded 86.96% accuracy for ocular surface disease detection, yet this performance dipped to 66.67% when tasked with pterygium grading under few-shot scenarios—a disparity that brings into sharp relief both the utility and the current friction points of LLM-driven workflows [68]. In a wider context, a systematic review aggregating data from 45,913 images across 20 studies reported impressive pooled sensitivity and specificity metrics (98.1%/99.1% for diagnosis). Nonetheless, the reliability of these findings is tempered by persistent methodological flaws: specifically, the scarcity of independent external validation, a tendency to rely on image-level rather than patient-level inference, and ambiguous decision thresholds. Resolving these structural weaknesses is non-negotiable before these systems can be safely embedded into routine clinical practice [69].

Current AI applications for pterygium have evolved to encompass smartphone-enabled screening, automated grading, segmentation, longitudinal monitoring, and outcome prediction. While performance metrics remain high across external tests, successful clinical translation hinges on establishing consensus grading definitions, conducting multicenter validation across diverse populations, and ensuring model interpretability through quantitative lesion metrics and saliency maps, alongside rigorous prospective impact assessment [58,59,63,64,69] (Table 4).

## 8. Fuchs Endothelial Corneal Dystrophy

Fuchs endothelial corneal dystrophy (FECD) is a common, age-related disorder of the corneal endothelium. In FECD, the endothelial cells degenerate and lead to corneal edema, glare, and visual impairment. Corneal transplantation remains the only treatment in the severe stage, so early detection is crucial [28,70]. However, the subtle changes in the endothelium decrease the accuracy of FECD diagnosis. This creates the opportunity for AI algorithms to assist ophthalmologists in detection and monitoring on images like specular microscopy (SM), widefield specular microscopy (WFSM), and AS-OCT.

SM has been widely utilized in DL-based models for corneal endothelium segmentation, corneal guttae analysis, and endothelial cell density (ECD) estimation. Various models have been developed for the test, including DenseUNets, U-Net, and Mobile-CellNet. DenseUNets with feedback non-local attention (fNLA) reduced errors in estimating ECD, the coefficient of variation (CV), and hexagonality (HEX) [71]. U-Net-based distance maps provided accurate division of ECD and cell area. These demonstrated the promise of AI approaches in detecting the corneal endothelium [72]. Mobile-CellNet achieved a mean absolute error (MAE) of 4.06% and required fewer computational resources in ECD estimation. Due to the accuracy and efficiency, Mobile-CellNet could be an emerging remote clinical assessment [73]. Collectively, these DL-based models provided precise and accurate results of corneal endothelium segmentation, supporting the development of remote FECD diagnosis.

Besides SM, there are also other images applied to DL-based segmentation, including IVCM and WFSM, for example, a fully automated system developed by IVCM for segmentation and morphometric parameter estimation, which achieved a high correlation with Topcon measurements [74]. A U-Net-based model with WFSM achieved a high Dice coefficient, which indicates consistency with manual segmentation. Moreover, it also indicated potential in assessing image quality and disease severity grading of FECD [75]. These studies highlighted the promise of AI algorithms in providing efficient and accurate assessment of FECD by reducing the limitations of ophthalmologists’ interpretation.

AI algorithms can also be employed in FECD automatic diagnosis and severity grading. For instance, DL-based models with AS-OCT images achieved high accuracy, sensitivity, and specificity in classification of early-stage and late-stage FECD and healthy corneas. The performance was combined with the clinical grading, which indicated the potential to assist experts in disease staging [76]. Similarly, a U-Net system provided precise evaluation of FECD through SM images and slit-lamp-based grading. The significant performance also increased the possibility of risk prediction and therapy monitoring [77]. More recently, enhanced compact convolutional transformer (ECCT) models with IVCM images achieved high accuracy and AUC in corneal endothelium disease (CED) detection. This highlighted the possibility of CED diagnosis in remote areas or during pandemics [78]. With the advancement of AI models, the errors of FECD detection, severity grading, and clinical decision-making can be decreased.

External validation has demonstrated the generalizability of AI algorithms for FECD detection. In the research, internal datasets achieved a higher AUC, sensitivity, and specificity than external datasets, indicating the limitation of automated FECD diagnosis. Recent studies of internal and external tests have shown trends in FECD detection. As the technique develops, the clinical application of FECD might increase [79].

Hybrid and advanced models shape the future of FECD assessment. A recent study combined multiple AI-based models and even traditional algorithms for FECD detection and segmentation. U-Net was applied with the Watershed algorithm to observe the changes in average perimeter length (APL), which reflected the severity of FECD. Moreover, many studies have combined a variety of datasets, such as slit-lamp images, SM, IVCM, WFSM, and AS-OCT, to grade the stage of FECD more accurately [77,80].

In the future, these trends may improve the accuracy of FECD early detection. Many experts have started to detect FECD via subclinical corneal edema, trying to apply AI models to clinical decision-making, treatments, and challenging cases [70,81] (Table 5).

## 9. Corneal Transplantation

AI algorithms have been integrated into corneal transplantation across preoperative, intraoperative, and postoperative phases [82]. The application includes predicting graft outcomes and guiding surgical decision-making through OCT and topography. AI-based models can improve the accuracy of different surgeries, such as penetrating keratoplasty (PK), deep anterior lamellar keratoplasty (DALK), Descemet stripping automated endothelial keratoplasty (DSAEK), and Descemet membrane endothelial keratoplasty (DMEK) [83].

During the preoperative phase, AI-based models have been applied to predict outcomes and guide surgical decision-making. For example, AS-OCT images were used in DL-based models to detect the risk of graft detachment and make the plans of surgeries. These models demonstrated high sensitivity in identifying detachment cases, thought the specificity still needed a lot of improvement. However, the AI approaches could analyze subtle differences between multiple images and provided the opportunity to perform precision surgery in corneal transplantation [84].

On the other hand, the application of AI approaches in postoperative phases has been more diverse. They could be employed to detect graft detachment, analyze risk factors, and predict the survival in long periods. ML models such as least absolute shrinkage and selection operator (LASSO), classification tree analysis (CTA), and random forest classification (RFC) achieved good performance in the AUROC of graft detachment prediction. A deep learning model was developed to localize and quantify graft detachment after DMEK, achieving high accuracy and producing quantitative detachment maps to assist clinical experts. For the long-term outcome, random survival forests and Cox regression were applied to predict 10-year graft survival for DSAEK and PK. The performance was much higher than traditional regression models. Overall, AI models not only improved the precision of surgeries, but also supported the management of treatment [85,86,87].

In summary, artificial intelligence demonstrates potential across in different phases of corneal transplantation. The systematic reviews also highlight that AI technologies can assist in candidate screening, surgical planning, and surgery monitoring. The diagnostic accuracy in preoperative and postoperative phases outperforms that of clinical experts. However, there are several challenges, including the absence of standard datasets, external validation sets, and studies of the aspects. In the future, experts can focus on multicenter datasets and hybrid AI-based models to improve the accuracy of AI models in corneal transplantation [88,89,90,91] (Table 6).

## 10. Discussion

Artificial intelligence shows great promise for addressing corneal conditions such as KC, DED, IK, pterygium, FECD, and corneal transplantation. Using various imaging approaches—including corneal tomography, meibography, AS-OCT, slit-lamp photos, IVCM, and even simple smartphone setups—AI systems regularly hit high marks in accuracy, sensitivity, and specificity, often matching or exceeding the efficiency and classification speed of human ophthalmologists [92,93]. In addition, these tools assist with classifying disease types, gauging severity, forecasting outcomes, and monitoring progression automatically, which aids physicians in making decisions and allows patients to take more control over their own treatment. Ultimately, this technology can support the entire patient care journey—from initial screening to long-term management—helping to expand access, especially in underserved regions.

Most reviews focused on the ability to diagnose, segment, grade, and predict by analyzing diagnostic metrics and models’ generalization. However, there are some limitations in previous reviews. For instance, Ji et al. (2022) primarily focused on diagnostic feasibility in KC, DED, IK, and pterygium and challenges in datasets [3]. Pagano et al. (2023) mainly provided an overview, with less emphasis on AI models [2]. Comprehensive studies like Nguyen et al. (2024) synthesized diagnostic performance metrics across various corneal conditions, while Nusair et al. (2025) advanced the field by advocating for reporting standardization (e.g., STARD-AI and QUADAS-AI checklists), and they also included the performance of LLMs [22,28]. In contrast to these works, our research addresses the functional gaps in CDSSs, especially in prescriptive prediction, and techniques like Federated Learning (FL) to solve ethical and privacy challenges. Moreover, we also provide a comparative analysis of different AI models across multiple diseases to guide future clinical integration.

Based on our review, distinct algorithmic architectures are suitable candidates for specific corneal diseases. Hybrid or multimodal fusion models with biomechanical data demonstrate increased performance over single-source CNNs in KC, especially in detecting subclinical cases. SimCLR has proven uniquely capable of identifying distinct clinical subtypes from meibography images in the management of DED. In IK, DenseNet121 exhibits superior performance in multiple studies in distinguishing between BK and FK. For pterygium screening, two-stage fusion models (like U-Net for segmentation) have proven robust even with smartphone-based imaging. In endothelial pathologies, segmentation accuracy for FECD is maximized by DenseUNets with feedback non-local attention, while computationally efficient models like Mobile-CellNet show promise for remote screening. Furthermore, for corneal transplantation prognosis, machine learning approaches such as random survival forests (RSFs) perform significantly better than traditional regression models in predicting long-term graft survival. This synthesis indicates that a single universal model might not be the best choice. The selection of AI models must be tailored to the specific imaging modality and clinical tasks.

Despite the impressive diagnostic metrics achieved in controlled settings, there are several challenges regarding data quality and availability. First, most studies have limited internal and external datasets, which may reduce model generalization across different populations and image conditions. In recent studies, more experts are integrating multiethnic data into training sets to deal with heterogeneity and reduce bias, thereby improving the performance of models in real-world settings [94,95,96,97,98]. Second, the heterogeneity of EHRs and data across institutions will influence the result of AI models. The implementation of standardized reporting guidelines, such as CONSORT-AI, could fix this problem. Additionally, AI models often struggle to train on limited datasets of rare corneal disease. Techniques such as data augmentation, generative modeling, and transfer learning can address these challenges [99,100].

Beyond data issues, the path to clinical translation is also limited by imaging standardization and validation. To address these problems, it is essential to establish standardized imaging protocols, preprocessing pipelines, and quality control. Crucially, the vast majority of current research lacks prospective validation. Randomized controlled trials (RCTs) are important to validate the AI models’ performance. RCTs can provide important evidence of AI performance in real-world clinical settings, assessing the impact on patient outcomes and decision-making processes. Nonetheless, the usage of RCTs is limited in ophthalmology because there are many challenges, such as high cost, prolonged duration of execution, lack of generalizability, and insufficient sample sizes, which limit the feasibility of RCTs on rare corneal disease. This indicates the need for alternative, rigorous prospective methodologies. Most studies rely on single-center datasets, highlighting the need for more prospective and multicenter trials [101,102,103].

There are also some gaps in CDSSs. The most critical deficiency is prescriptive prediction. While recent models can only analyze data and grade the disease, they mostly fail to propose specific interventions and therapeutic responses. For example, current models may accurately detect IK but fail to predict the response to specific antimicrobial agents or recommend the optimal timing for therapeutic keratoplasty. Additionally, existing models struggle to integrate genetics, lifestyle factors, and environmental factors to predict the long-term prognosis and risk stratification. Finally, dynamic medication alerting is also an unresolved issue. Current systems lack the capability to analyze ocular findings with systemic health profiles or medication history derived from EHRs [104].

While AI can help clinical decision-making, the role of physicians still cannot be replaced. AI serves as a support tool for clinicians to detect disease more accurately. However, specialists are expected to interpret AI results carefully to ensure the appropriate clinical decision and treatment. Currently, AI models are predominantly image-based, differing from the comprehensive diagnostic process of corneal diseases. In corneal management, clinical history, symptom severity, fluorescein staining patterns, anterior chamber reaction, pain level, and disease progression are crucial influences on decision-making. These limit AI’s ability to incorporate treatment urgency, progression, or specific management recommendations. Although the early detection and grading of disease is improved, the final interpretation and decision-making still rely on ophthalmologists’ judgment.

Beyond those functional limitations, the hurdles for clinical adoption also include trust and accountability. The black-box problem and critical ethical concerns increase the difficulties of AI application. The majority of deep learning models lack explanation of their complex decision-making process, which causes the “black-box” problem [105]. The lack of transparency is a challenge for experts to understand the behavior, identify potential biases, and check the errors of AI models. Recently, XAI and model visualization have been developed to address the issue. XAI can provide visual evidence and transparency regarding the explanation in clinical practice, allowing clinicians to analyze the results and verify the model’s clinical plausibility [106,107]. Moreover, data privacy and security are also important ethical issues. While training AI models, large numbers of images or data are the main resources. Those data should comply with strict regulations such as Health Insurance Portability and Accountability (HIPAA) or General Data Protection Regulation (GDPR). These regulations regulate the collection, storage, and sharing of the datasets. However, multicenter data is important for the internal and external tests, which enhance models’ generalization. Therefore, the protection of shared private data, such as through FL, differential privacy, hybrid privacy-preserving techniques, and cryptographic techniques, is very important [108,109].

In the future, multicenter and prospective clinical trials will apply AI technologies to detect and manage corneal diseases [98,110,111]. To bridge the current functional gaps in CDSSs, future research must prioritize the development of multimodal fusion architectures. Experts could add other information such as symptoms, history, and laboratory results into the models. Regulatory frameworks are also evolving to facilitate the rules to regulate AI-based tools. For example, the Food and Drug Administration (FDA) approved the first AI device for diabetic retinopathy in 2018, demonstrating that regulatory pathways for clinical AI exist. Through XAI, robust datasets, and multimodal fusion, AI tools can be changed from a diagnostic aid into a comprehensive clinical helper in corneal healthcare.

## 11. Conclusions

While AI models show great promise for diagnosis, their common use in clinics depends on fixing data biases and proving their performance through strict real-world testing. To close current gaps, future systems must evolve into multimodal CDSSs that combine patient history with images, along with privacy protections to ensure ethical data use. Ultimately, the goal of AI is to support the ophthalmologist, turning technical precision into a trusted, essential partner for patient care.

## Figures and Tables

**Table 1:** AI models applied to keratoconus.

Study	AI Model	Objective	Data (*N*)	Dataset Availability	Test Split (*N*)	Main Outcomes
Ghasedi et al. (2025) [26]	DL + Genetic Algorithm	KC and suspect detection	Pentacam (*N* = 1288 eyes)	Private	3-fold cross-validation	Optimized feature selection using Genetic Algorithms (GAs) significantly improved diagnostic accuracy by focusing on relevant features. (ANN + GA: Acc = 98.63%; SVM + GA: Acc = 98.13%; ANN alone: 96.9%.)
Alió Del Barrio et al. (2024) [20]	ANN (MLP)	KC and suspect KC detection	AS-OCT and Placido topography (*N* = 6677 eyes)	Private	30% of dataset	Early detection and automated clinical support of KC; the model outperformed in KC, but the Recall of suspect KC was low. (KC: Prec = 96.0%, Rec = 97.9%, F1 = 96.9%; Suspect: Prec = 83.6%, Rec = 69.7%, F1 = 76.0%)
Abdelmotaal et al. (2024) [21]	CNN (DenseNet121)	KC detection	Corneal deformation videos (*N* = 734 eyes)	Private	Dataset 2: 30%; dataset 1: external validation	AUROC = 0.93, showed generalization in external test; a quick and objective tool because only needs Corvis ST
Hartmann et al. (2024) [23]	DL	Predict KC progression	Pentacam clinical data (*N* = 570 KC eyes)	Private	10%	Predicts progression at the first visit, helping early CXL decisions; fusion of imaging and numerical data is better. (Fusion Model Acc = 0.83; Imaging only Acc = 0.77)
Ambrósio et al. (2023) [25]	Random forest (TBIv1/v2)	Ectasia detection	Pentacam + Corvis (*N* = 3886 eyes)	Private	10-fold cross-validation	Improve the detection of subclinical ectasia. (Very asymmetric ectasia, VAE-NT 0.899 to 0.945)
Lu et al. (2023) [19]	Random forest and NN	FFKC detection	Pentacam + OCT + Corvis ST (*N* = 599 eyes)	Private	40% validation	Multiple devices are better; found that combining biomechanics (Corvis) with OCT was sufficient (AUC 0.902), with no added benefit from a 3rd device. (Corvis + OCT AUC = 0.902; Three-device AUC = 0.871)
Maile et al. (2022) [24]	Royston–Parmar	Predict KC progression	Keratometry and pachymetry (*N* = 8701 eyes) Genetic data (*N* = 926 patients)	Private	Internal–external cross-validation by region	First integration of genetic scores with topography; addresses biological risk factors. (Explained variation 33%; age was the most significant predictor)
Chen et al. (2021) [4]	Deep learning (VGG16)	KC detection and staging	Colour-coded maps (*N* = 1926 scans)	Private	*N* = 532	Validated a CNN model on multicenter datasets using colour-coded maps; achieved high accuracy in external validation. (Testing Acc = 97.85%; external Val AUC = 0.9737)
Elsawy et al. (2021) [18]	Multi-disease CNN	Multidisease diagnosis (KC, FED, DES)	AS-OCT (*N* = 158,220)	Private	*N* = 132 eyes	MDDN enables automated screening for KC alongside other corneal diseases (FED, DES) using only AS-OCT, demonstrating high clinical utility for comprehensive triage. (Eye-level AUROC > 0.99 for KCN; F1 score > 0.90)
Herber et al. (2021) [12]	ML–linear discriminant analysis (LDA), random forest (RF)	Classifying the stage of KC	Dynamic Scheimpflug tonometry (Corvis ST) (*N* = 434 eyes)	Private	30% Validation	The CST can predict the stage of KC without keratometric data; classification based on biomechanical properties. (Overall KC detection Acc = 93%)
Kamiya et al. (2019) [17]	Deep learning (VGG16)	Evaluate accuracy of KC diagnosis	AS-OCT (*N* = 543 eyes)	Private	LOOCV (5-fold)	AS-OCT maps effectively discriminate KC stages; increase accuracy in daily practice. (Normal vs. KC Acc = 0.991; stage classification Acc = 0.874)
Lavric et al. (2019) [16]	CNN (KeratoDetect)	Automatic detection of KC	Placido topography (*N* = 3000 images)	Private	*N* = 400	Rapid screening tool; assists ophthalmologists to detect. (Accuracy 99.33% on test set)
Ruiz et al. (2016) [11]	ML-SVM	Evaluate the performance of SVM in detection	Pentacam (*N* = 856 eyes)	Private	no specified	Comparison with single parameter method; objective KC detection. (5-group accuracy 88.8%, Sens = 90%, Spec = 95.2%)
Smadja et al. (2013) [10]	ML-Regression tree	Detection of subclinical KC	Placido and Scheimpflug (*N* = 372 eyes)	Private	Not specified	Help in surgical decision and detect early KC. (Normal vs. FFKC: Sens = 93.6%, Spec = 97.2%)
Arbelaez et al. (2012) [9]	ML-SVM	Define ML-based classification	Placido tomography (*N* = 3502 eyes)	Private	Validation set	Classification of subclinical KC detection. (Subclinical KC Sens: 75.2% improved to 92%)
Accardo et al. (2002) [15]	Neural network	Screening for KC detection in both eyes	Videokeratoscope (EyeSys, *N* = 396 eyes)	Private	Not specified	Using parameters from both eyes improves KC detection; supports early detection and screening. (Test set: Global Sens 94.1%, Global Spec 97.6%)
Smolek et al. (1997) [14]	Neural network	Detection and grading stage of KC or KCS	Videokeratoscope TMS-1 (*N* = 300 eyes)	Private	*N* = 150	Distinguish KC and KCS; improves accuracy and specificity over conventional tests. (Classification network: 100% accuracy, sensitivity, specificity)
Maeda et al. (1995) [8]	Neural network	Automatically distinguishing topography	videokeratoscope TMS-1 (*N* = 183 eyes)	Private	*N* = 75	Reduce objective interpretation; support diagnosis of corneal shape abnormalities. (Test: 80% accuracy; accuracy and specificity >90% for all categories)
Maeda et al. (1994) [7]	Expert system	Differentiate keratoconus	TMS-1 (*N* = 200 eyes)	Private	*N* = 100	Reduce subjective opinion of topography interpretation. (Validation: Acc = 96%, Spec = 99%, Sens = 89%)

**Table 2:** AI models applied to dry eye disease (DED).

Study	AI Model	Objective	Data (*N*)	Data Availability	Testing Split (*N*)	Main Outcomes
Zhang et al. (2025) [36]	Mask R-CNN (ResNet-101)	Tear meniscus height (TMH) measurement	Ocular surface images (*N* = 1300)	private	*N* = 220 (internal = 120, external = 100)	Achieved high precision with IoU 0.928 and *R*^2^ 0.92, with external validation AUC reaching 0.975; limited sample size led to potential selection bias and unequal distribution of dry eye severity
Wang et al. (2025) [37]	ALNN (Attention-Limiting NN based on U-Net)	Automatic TMH measurement	Ocular surface images (*N* = 1300)	private	*N* = 2536	Demonstrated superior segmentation (MIoU > 0.92) across multicenter data, with color images performing better than infrared (*r* = 0.957 vs. 0.803); scarcity of data with high TMH (>0.4 mm) reduces robustness for severe cases; method has device-specific dependency on Keratograph 5M
Nejat et al. (2024) [39]	Deep learning (SimCLR NN)	Tear meniscus height measurement	Smartphone images (*N* = 1021)	private	not specified	Achieved Dice coefficient of 0.9868 and accuracy of 95.39% using accessible smartphone imagery; limited to specific smartphone optics; lack of large-scale external validation
Li et al. (2023) [31]	Deep learning (SimCLR NN)	Clustering of dry eye subtypes	Meibography images (*N* = 82,236)	private	no	Identified 6 distinct subtypes (unsupervised) with TBUT and TMH; if no image subtypes, detailed clinical validation was limited to a small subset of 280 patients
Shimizu et al. (2023) [33]	CNN–Swin Transformer	Tear film break-up time (TFBUT) estimation for DED	Smart Eye Camera videos (slit-lamp video frame) (*N* = 22,172 frames)	private	1599 frames	Achieved TFBUT estimation accuracy of 78.9% and DED diagnosis AUC of 0.813, enabling portable screening. Annotation relied on a single specialist, causing potential bias; strict image quality filtering limits real-world utility; lacked validation across different races. (TFBUT: Acc = 78.9%, AUROC = 0.877, F1 = 0.74; DED (ADES): Sens = 77.8%, Spec = 85.7%, AUROC = 0.813)
Qu et al. (2023) [38]	ResNet-34 + U-Net	Punctate Epithelial Erosion (PEE) grading	Slit-lamp corneal fluorescein staining images (*N* = 763)	private	20% split	Achieved grading accuracy of 76.5% (AUC 0.940) and segmentation IoU of 0.937, showing high correlation with clinical grades (*r* = 0.908). (Gap: single-center study with single ethnic background; non-linear NEI scale limits objective comparison; required high-quality images with 283 excluded)
Chase et al. (2021) [34]	DL-VGG19	DED diagnosis using AS-OCT	AS-OCT images (*N* = 27,180)	private	*N* = 7020	Diagnostic agreement significantly better than Schirmer’s test and staining (84.6% accuracy); lack of age-matching standard for DED definition affects ground truth; model cannot quantify severity
Yeh et al. (2021) [30]	NPID (Unsupervised ResNet-50)	Meibography phenotyping	Meiboscore and infrared meibography images (*N* = 706 images)	private	*N* = 209	Achieved TFBUT estimation accuracy of 78.9% and DED diagnosis AUC of 0.813, enabling portable screening. Source: Infrared meibography images (*N* = 706 images)
Wang et al. (2019) [29]	PSPNet/DeepLab	Meibomian Gland (MG) atrophy segmentation	Meiboscore and infrared meibography images (*N* = 706)	private	*N* = 209	Achieved high segmentation accuracy (eyelid 97.6%, atrophy 95.4%) and Meiboscore grading accuracy of 95.6%. Cannot analyze individual gland morphology such as tortuosity or thickness; limited dataset size compared to later studies
Su et al. (2018) [32]	CNN	TFBUT measurement	Slit-lamp fluirescein tear film video (*N* = 80 patient)	private	*N* = 30	Demonstrated high correlation with manual TFBUT (*r* = 0.9) and achieved sensitivity 0.83/specificity 0.95 for screening; training set is too small; only right eyes analyzed; manual ROI selection required; sensitive to eye movements and blinking

**Table 3:** AI models applied to infectious keratitis (IK).

Study	AI Model	Objective	Data (*N*)	Dataset Availability	Test Split (*N*)	Main Outcomes
Li et al. (2024) [50]	Stage1: DL-CNN (Swin Transformer); Stage2: Multi-instance learning+ attention	DL diagnoses FK	IVCM; *N* = 96,632	private	stage 1 IVCM (*N* = 8568); stage 2 = 37 patients	Spec. = 96.65%, Sens. = 97.57%; two stages can improve specificity and sensitivity; second stage mimics the clinical workflow and reduces wrong diagnose of FK cases
Erukulla et al. (2025) [51]	DL- ResNet50	Model 1: Classify Fk, AK, NSK; Model 2: FK is filamentous or non-filamentous	IVCM (*N* = 1975)	private	formed through rotating folds in cross-validation	Accurately classify IK and subtype FK (multi-class diagnosis); accuracy > 85% in both models; Grad-CAM can visualize regions to influence prediction
Soleimani et al. (2025) [52]	DL-CNNs	DL diagnoses different kinds of MK by smartphone	Smartphone images (*N* = 602)	private	20% of dataset	Accuracy 83.8%; discrimination accuracy of AK, BK, FK is higher than 0.80; by using smartphone and slit-lamp adaptor, solve the gaps in the remote environment
Satitpitakul et al. (2025) [47]	CNN (DenseNet121, ResNet50, VGG19)	Differentiate IK	Slit-lamp images (*N* = 6478)	private	*N* = 1307	DenseNet121’s accuracy in four-class classification *N* = 0.80; ensemble algorithm’s accuracy = 0.83, better than a single tool; potential in resource-limited countries
Li et al. (2024) [53]	DeepIK (DenseNet121, InceptionResNetV2, Swin Transformer)	Identify BK, FK, AK, NSK	Slit-lamp images (*N* = 23,055)	private	(*N* = 12,463)	AUC: internal test 0.95–0.99, external test 0.88–0.93, prospective 0.87–0.97; achieve high AUC in external dataset, which ensures the generalizability
Essalat et al. (2023) [42]	CNN (DenseNet161, DenseNet121, etc.)	Automated diagnosis of FK and AK	IVCM images (*N* = 4001)	public (Figshare)	*N* = 1001	Densenet161 achieved 93.55% accuracy, potential in FK and AK early detection with eXplainable Artificial Intelligence (XAI)
Wei et al. (2023) [43]	ML (Logistic, RF, DT)	Differentiate FK	Slit-lamp images (*N* = 1916)	private	internal *N* = 449; external *N* = 420	First machine learning model for FK diagnosis; AUC of internal and external is over 0.90, which supports clinical decision-making
Kuo et al. (2021) [44]	DL (ResNet, DenseNet, ResNeXt, SE-ResNet, and EfficientNets)	Different DL algorithms for classifying BK	Slit-lamp (*N* = 1512)	private	20% of each fold in 5 fold cross-validation	Comparing DL models and best AUROC is EfficientNet B3; EfficientNets were lesion focused without segmentation and preprocessing
Soleimani et al. (2023) [45]	CNN	Diagnose IK (model 1), differentiate BK and FK (model 2), discriminate filamentous type from yeast type of fungal (model 3)	Slit-lamp images (*N* = 9329)	private	have testing split	Accuracy: Model 1: 99.3%; Model 2: 84%; Model 3: 77.5%; assist experts to distinguish species of IK; first DL model of yeast and filamentous fungi
Tang et al. (2023) [48]	DL and DT	Classification of FK and AK	IVCM(*N* = 3364)	private	*N* = 334	DL performs better than DT; classification of Fusarium and Aspergillus; real-time, non-invasive test; guide for treatment
Liang et al. (2023) [49]	DL-CNN (GoogLeNet and VGGNet)	Diagnosis of FK	IVCM (*N* = 7278)	private	(*N* = 1455)	Accuracy = 97.73%; support faster FK diagnosis; incorporate simple prior knowledge of hyphae-like structures to enhance accuracy
Hanif et al. (2023) [56]	DL-CNN	Evaluate how corneal images’ quality affect CNN prediction	DSLR	private	*N* = 3307 (External Test)	AUROC: FK = 0.85, BK = 0.79, micro-averaged = 0.83; supports AI diagnosis; resource-limited setting
Li et al. (2021) [46]	DL- DenseNet121, Inception-v3, ResNet50	Classify keratitis	Slit-lamp and smartphone (*N* = 13,557)	private	have testing split	DenseNet121 is the best on every dataset; comparable to specialists in early detection
Kuo et al. (2020) [54]	DL- DenseNet	Comparison of FK diagnosis between AI models, experts and NCS-Oph	DSLR (*N* = 288)	private	not specified	Primary care, specificity higher than NCS-Oph (Sens: DL71% > NCS-Oph 52%; Spec: DL68% < NCS-Oph 83%)
Liu et al. (2020) [55]	DL-CNN (AlexNet, VGG16)	Detection of FK	IVCM (*N* = 1213)	private	1/11 datasets	Improves real-time diagnostic performance; reduces reliance on expert subjective judgment (accuracy: AlexNet 99.95%, VGGNet 99.89%)

**Table 4:** AI models applied to pterygium.

Study	AI Model	Objective	Data (*N*)	Data Availability	Testing Split (*N*)	Main Outcomes
Li et al. (2025) [68]	Multimodal Ocular Surface Assessment and Interpretation Copilot (MOSAIC)	Detection and grading of ocular surface diseases (OSDs)	Smartphone images (*N* = 375)	private	*N* = 375	Improve the capability of image comprehension (ROUGE-L F1 scores of 0.70–0.78); limited dataset diversity; risk of LLM hallucinations; gap remains between research and product implementation
Zhang et al. (2024) [65]	Weighted correlation network analysis (WGCNA), RF, SVM	Classifying molecular mechanisms for detecting pterygium	RNA-seq (*N* = 68)	public (NCBI’s Sequence Read Archive and Gene Expression Omnibus)	Validation on external datasets (GSE2513, GSE51995)	Modest sample size limits generalizability; lack of long-term clinical follow-up for prognosis; potential batch effects in sequencing; need for experimental validation of causative relationships
Liu et al. (2024) [63]	Faster R-CNN (ResNet101) + U-Net (SE-ResNeXt50)	Pterygium detection and grading	Slit-lamp images (*N* = 20,987) + smartphone (*N* = 1094)	private	slit-lamp (*N* = 6296) + smartphone (*N* = 329)	Fusion model on smartphone images can be comparable to slit-lamp (accuracy of 92.38%, F1 = 0.931); image lacks medical history; need auto-QC to select eligible images; cannot identify other diseases
Gan et al. (2022) [60]	DL-ResNet18, AlexNet, GoogleNet, VGG11	Diagnosis and treatment of pterygium	Anterior segment images (*N* = 172)	private	*N* = 34	Ensemble model performs better than single models (AUC = 0.98); triage for surgery; proposes use in underserved settings; small size of data limits generalizability; black-box problem
Fang et al. (2022) [64]	VGG16 (ImageNet) and MLP	Referable pterygium	Slit-lamp/hand-held images (*N* = 2503), internal and external sets	private	internal (*N* = 629); external 1 (*N* = 2610), external 2 (*N* = 3701)	AUC of referable pterygium reached 98.5%; community screening feasibility; limited positive referable cases in external tests
Hung et al. (2022) [66]	Deep learning system (DLS)	The efficacy of DL in grading and prediction	Slit-lamp images (*N* = 237 eyes)	private	*N* = 48 eyes (grading), *N* = 25 (recurrence)	Early evidence; needs larger, prospective validation
Wan et al. (2022) [62]	U-Net++	Diagnose and measure pterygium	AST (*N* = 489)	private	*N* = 239	Objective tracking of lesion progression for follow-up; relied on doctors’ visual inspection
Zhu et al. (2022) [61]	VGG16 (screening) and PSPNet (segmentation)	Screening and lesion segmentation	Slit-lamp images (*N* = 734)	private	screening (*N* = 300), segmentation (*N* = 150)	Masks for corneal encroachment; aids pre-op planning (screening accuracy = 99%, segmentation MIOU = 0.86, best among models); need larger datasets to achieve better sensitivity
Jais et al. (2021) [67]	SVM, DT, Logistic Regression	Prediction of BCVA changes	Clinical dataset (*N* = 93)	private	10-fold cross validation	Decision support for postoperative counseling (specificy= 100%); small size of sample influences the result; black-box nature

**Table 5:** AI models applied to Fuchs endothelial corneal dystrophy (FECD).

Study	AI Model	Objective	Data (*N*)	Data Availability	Testing Split	Main Outcomes
Tey et al. (2024) [58]	DL(U-Net)	Evaluate DL in FECD analysis	Widefield specular microscopy (*N* = 1839)	private	external dataset (*N* = 354)	Novel application on widefield imaging to assess peripheral endothelium (Dice coefficient in DL vs. manual = 0.86, Central ECD DL higher than manual); eye without clinically definite edema
Fitoussi et al. (2024) [81]	DL	Detect subclinical corneal edema in FECD	Optical coherence tomography (OCT) (*N* = 151)	private	*N* = 151 (validation cohort)	Can detect subclinical edema in atypical cases; small sample size and false positive in the datasets (model and tomography features agreed in 80% of cases)
Qu et al. (2024) [78]	DL-Enhanced Compact Convolutional Transformer (ECCT)	Establish automatic diagnosis system for corneal endothelium diseases (CEDs)	IVCM (*N* = 3723)	private	multicentre (*N* = 449)	First AI system for multiple CEDs using IVCM (accuracy = 89.53%, AUC = 0.958); sensitivity of “others” is low, so needs larger datasets for rare disease
Prada et al. (2024) [77]	DL-CNN (U-Net)	Classification of FECD severity	Specular microscopy and slit-lamp images (*N* = 1371)	public (Open Science Framework)	not specified	Significant difference in cell density between AI-based and built-in software
Foo et al. (2024) [79]	DL(+RF)	Diagnosis of FECD	Specular microscopy (internal and external sets)	private	external (*N* = 180), peripheral (*N* = 557)	First DL model for the peripheral endothelium; performance drop in external (1st model: AUC = 0.96, Sens. = 0.91, Spec. = 0.91 (internal); AUC = 0.77, Sens. = 0.69, Spec. = 0.68 (external))
Sierra et al. (2023) [72]	Deep learning (U-Net)	Segment corneal endothelium and guttae in FECD	Specular microscopy (*N* = 90)	private	*N* = 23	Cast segmentation as a regression task (mean ECD difference = −41.9 cells/mm^2^; mean cell area difference = 14.8 µm^2^); requires larger datasets
Karmakar et al. (2023) [73]	Mobile-CellNet, U-Net, U-Net++	Automatic segmentation and ECD estimation	Specular microscopy (*N* = 612)	public	holdout set (*N* = 124)	A lightweight model suitable for remote settings (mean absolute error: ECD = 4.06%, U-Net = 3.80% and FLOPs less); comparison is limited to visual inspection for some failure cases
Vigueras et al. (2022) [71]	Deep learning (U-Net, ResUNeXt, DenseUNets)	Automated segmentation and morphometric analysis of corneal guttae	specular microscopy (*N* = 1203)	private	10-fold cross-validation	Novel feedback non-local attention to infer cell edges;inference of edges inside large guttae is probabilistic (MAE: ECD = 23.16 cells/mm^2^; CV = 1.28%; HEX = 3.13%)
Qu et al. (2022) [74]	DL (ResNet)	Automated segmentation and morphometric parameter estimation system	IVCM (*N* = 283)	private	*N* = 184	First fully automated DL system with IVCM; longitudinal studies not possible with IVCM; (ECD: 2592 cells/mm^2^; CV: 32.14%; HEX: 54.16%)
Shilpashree et al. (2021) [80]	DL (U-Net) and Watershed algorithm	Segment FECD and healthy patients	Specular microscopy images (*N* = 246)	private	*N* = 246	U-Net and Watershed to resolve merged cells; average perimeter length (APL) as a new biomarker; APL only apparent after 5% of guttae
Eleiwa et al. (2020) [76]	DL-VGG19	Automatic diagnosis of early-FECD, late-FECD, and healthy cornea	AS-OCT (*N* = 187,20)	private	*N* = 7380	First diagnosis by AS-OCT; although large number of pictures, only 81 patients (early-FECD: AUC 0.997; healthy vs. all FECD: AUC 0.998)

**Table 6:** AI models applied to corneal transplantation.

Study	AI Model	Objective	Data (*N*)	Data Availability	Testing Split (*N*)	Main Outcomes
Patefield et al. (2023) [84]	DL-ResNet (MIL-AI)	Distinguish the graft detachment on pre-DMEK scans	AS-OCT; (*N* = 9466)	private	*N* = 24 (eyes)	First AI model to predicts eye with post-DMEK detachment; opportunity for DMEK screening; small data size (*N* = 74 eyes) cause black-box problem. (AUROC = 0.6; Sens = 92%, Spec = 45%)
Muijzer et al. (2022) [85]	ML–LASSO, classification tree analysis (CTA), random forest classification (RFC)	Prediction of graft detachment after posterior lamellar keratoplasty	Multimodal clinical data (*N* = 3647)	private	30% of the spilt	Identify key risk factors and protective factors; model cannot explain all variance (AUROC: LASSO = 0.7, CTA = 0.65, RFC = 0.72)
Ang et al. (2022) [87]	ML–random survival forests (RSFs) and Cox regression	Analyze factors affecting 10-year graft survival in DSAEK and PK	Multimodal clinical data (*N* = 1335)	private	out-of-bag validation	confirmed long-term superiority of DSAEK over PK in Asian eyes (10-year graft survival: DSAEK 73.6% vs. PK 50.9%); specific to Asian population
Heslinga et al. (2020) [86]	DL (U-Net and ResNet)	Automatically locate and quantify graft detachment after DMEK	AS-OCT (*N* = 1280)	private	*N* = 320	Quantify detachment length and visualize on 2D maps; support DMEK research; help clinical decision-making; limit generalization (Dice score: segmentation model = 0.896 vs. experts = 0.880)

## Data Availability

No new data were created or analyzed in this study. Data sharing is not applicable to this article.

## Abbreviations

The following abbreviations are used in this manuscript:

AI	Artificial intelligence
DED	Dry eye disease
IK	Infectious keratitis
MeSH	Medical Subject Headings
ML	Machine learning
DL	Deep learning
ANN	Artificial neural network
CNN	Convolutional neural network
DNN	Deep neural network
AS-OCT	Anterior segment optical coherence tomography
KC	Keratoconus
FFKC	Forme fruste keratoconus
SVM	Support vector machine
LDA	Linear discriminant analysis
RF	Random forest
DT	Decision tree
NNs	Neural networks
TVST	Translational Vision Science and Technology
AUC	Area Under the Curve
AUROC	Area Under the Receiver Operating Characteristic Curve
CDSSs	Clinical Decision Support Systems
XAI	eXplainable Artificial Intelligence
EHRs	Electronic Health Records
MGD	Meibomian gland disease
TMH	Tear meniscus height
TBUT	Tear film break-up time
OCT	Optical coherence tomography
ADES	Asia Dry Eye Society
OSDI	Ocular surface disease index
IVCM	In vivo confocal microscopy
FK	Fungal keratitis
BK	Bacterial keratitis
AK	Acanthamoeba keratitis
MK	Microbial keratitis
VGG	Visual Geometry Group
DSLR	Digital single-lens reflex
BCVA	Best-corrected visual acuity
LLM	Large language model
FECD	Fuchs endothelial corneal dystrophy
SM	Specular microscopy
WFSM	Widefield specular microscopy
ECD	Endothelial cell density
fNLA	Feedback non-local attention
CV	Coefficient of variation
HEX	Hexagonality
MAE	Mean absolute error
ECCT	Enhanced compact convolutional transformer
CEDs	Corneal endothelium diseases
APL	Average perimeter length
PK	Penetrating keratoplasty
DALK	Deep anterior lamellar keratoplasty
DSAEK	Descemet stripping automated endothelial keratoplasty
DMEK	Descemet membrane endothelial keratoplasty
LASSO	Least absolute shrinkage and selection operator
CTA	Classification tree analysis
RFC	Random forest classification
FL	Federated Learning
RSFs	Random survival forests
RCTs	Randomized controlled trials
HIPAA	Health Insurance Portability and Accountability
GDPR	General Data Protection Regulation
FDA	Food and Drug Administration
Sens	Sensitivity
Spec	Specificity
NCS-Ophs	Non-consultant specialist ophthalmologists
